# Long-term outcomes in Primary congenital glaucoma, aniridia and anterior segment dysgenesis

**DOI:** 10.1177/11206721211073208

**Published:** 2022-01-10

**Authors:** Tejal Magan, Alexander Tanner, Julia Fajardo-Sanchez, Kin Sheng Lim, Saurabh Goyal, Ian Rodrigues, Luis Amaya, Sameer Trikha, Avinash Kulkarni, Christopher Hammond, Gerassimos Lascaratos, Cynthia Yu-Wai-Man

**Affiliations:** 1 Faculty of Life Sciences & Medicine, 111990 King’s College London, London, UK; 2 Department of Ophthalmology, 111988 St Thomas’ Hospital, London, UK; 3 Department of Ophthalmology, 111990 King’s College Hospital, London, UK

**Keywords:** Primary congenital glaucoma, aniridia, anterior segment dysgenesis, glaucoma surgery

## Abstract

**Aim:**

To determine the long-term outcomes of a cohort of complex patients with primary congenital glaucoma, aniridia and anterior segment dysgenesis.

**Methods:**

Retrospective consecutive series between 1990–2021 in two UK tertiary centres: Guy's and St Thomas’ NHS Foundation Trust and King's College Hospital NHS Foundation Trust. We recorded the number and types of surgical and laser treatments along with preoperative and postoperative data, including intraocular pressures (IOP) and anti-glaucoma medications.

**Results:**

A total of 41 eyes of 21 patients were included. Primary diagnoses were primary congenital glaucoma in 16 eyes (39.0%), aniridia in 14 eyes (34.2%), and anterior segment dysgenesis in 8 eyes (19.5%). Sixteen eyes (39.0%) had one or more glaucoma surgery or laser procedures for advanced glaucoma, and the long-term follow-up was 12.8 ± 3.6 years. There was a significant decrease in postoperative IOP (mmHg) at 3 months (16.5 ± 1.6; *p* = 0.0067), 6 months (18.7 ± 2.1; *p* = 0.0386), 12 months (18.6 ± 1.7; *p* = 0.0229), 3 years (14.7 ± 1.2; *p* = 0.0126), 5 years (15.5 ± 1.8; *p* = 0.0330) and 10 years (15.4 ± 2.3; *p* = 0.7780), compared to preoperatively (24.1 ± 2.6). Surgical success (complete and qualified) was 62.5%, 50.0%, 43.8%, 46.2%, 45.5% and 28.6% at 3 months, 6 months, 12 months, 3 years, 5 years and 10 years, respectively. There was no significant change in the number of anti-glaucoma drugs postoperatively (*p* > 0.05). Four eyes (25.0%) had postoperative complications (hyphaema, hypotony) that resolved after conservative management.

**Conclusions:**

Surgical management of these complex eyes with advanced glaucoma is challenging. Overall, the cohort had good surgical outcomes with a significant decrease in IOP by 36.1% after long-term follow-up.

## Introduction

Childhood glaucoma is classified as primary or secondary.^1^ Primary childhood glaucoma is due to developmental abnormalities of the angle and comprises primary congenital glaucoma (PCG) and juvenile open-angle glaucoma. Secondary childhood glaucoma results from a reduction in aqueous outflow due to a condition that is acquired after birth or is present at birth (non-acquired). This is associated with conditions like anterior segment dysgenesis (ASD), aniridia, Sturge-Weber syndrome (SWS) and aphakia.

PCG has an incidence of 1: 18,500 in the UK,^2^ 1: 38,000 in Spain,^3^ 1: 30,000 births in Australia,^4^ 1: 3,300 in the southern state of Andhra Pradesh (India),^5^ 1: 2,500 in Saudi Arabia,^6^ 1: 68,254 in Minnesota (USA),^7^ and 1: 1,250 in individuals of Slovakian Roma descent.^8^ High prevalence is also typically observed in populations with higher levels of parental consanguinity.^9^ These rare cohorts of patients are often complex and their glaucoma is difficult to manage surgically. Owing to the rarity, there are only a small number of published cohorts and there is limited data available on the long-term surgical outcomes in these conditions.

Our study aims to describe the long-term surgical outcomes of a cohort of complex glaucoma patients with PCG, aniridia, ASD or SWS in two UK tertiary referral centres: Guy's and St Thomas’ NHS Foundation Trust and King's College Hospital NHS Foundation Trust.

## Materials and methods

### Data collection

A retrospective consecutive series was carried out in the glaucoma service for those patients who had a diagnosis of PCG, aniridia, ASD or SWS made at any point between 1990–2021 in two UK tertiary referral centres: Guy's and St Thomas’ NHS Foundation Trust and King's College Hospital NHS Foundation Trust. Medical record systems, which included both electronic and paper records, were reviewed for these patients in both trusts.

The data were collected with no identifiable patient details, and all patients who had anterior segment or fundus photography had consented for their images to be used for research and teaching purposes. The data protection act was adhered to for the protection of patient data and all investigations were undertaken in accordance with the declaration of Helsinki.

### Clinical examination

Detailed clinical data were collected from the medical records, including the diagnosis and type of glaucoma, best-corrected visual acuity, intraocular pressure (IOP) and cup-to-disc ratio. IOP was measured in clinic using the iCare tonometer in children and using the Goldmann applanation tonometer in adults. We also collected detailed information on patient demographics, including age, gender and ethnicity. Anterior segment photographs of patients were taken using a slit lamp camera (Keeler, UK). Dilated fundus photography and optical coherence tomography (OCT) disc imaging of the retinal nerve fibre layer thickness were also performed using the DRI-OCT Triton machine (Topcon, UK).

### Surgical and medical treatment

We recorded the number and types of glaucoma surgeries and laser treatments, including goniotomies, trabeculotomies, trabeculectomies, glaucoma tube surgeries and cyclodiode laser treatments. Details of other ocular (non-glaucoma) surgeries were also recorded. Detailed clinical data were collected preoperatively and postoperatively, including IOP and the number of anti-glaucoma medications.

Primary outcome was defined as complete success (6 ≤ IOP≤21 mmHg with ≥20% reduction from baseline, no anti-glaucoma medication), qualified success (same parameters as in complete success plus anti-glaucoma medication), or failure (IOP>21 mm Hg or not reduced by 20%; IOP≤5 mm Hg with vision loss on two consecutive visits; reoperation for glaucoma; or loss of light perception vision).

### Statistical analyses

All results represent mean and standard error of the mean. To determine statistical significance, a Student's t-test was performed and *p* values were calculated. We used Kaplan Meier survival probability graphs for complete success and qualified success. *p* < 0.05 was considered to be statistically significant.

## Results

### Patient demographics

A total of 41 eyes of 21 patients, who had a diagnosis of PCG, aniridia, ASD or SWS, were identified from the glaucoma service at Guy's and St Thomas’ NHS Foundation Trust and King's College Hospital NHS Foundation Trust. The demographics of the patient cohort are detailed in Table 1. The most common diagnosis was PCG in 16 eyes (39.0%), followed by aniridia in 14 eyes (34.2%), ASD in 8 eyes (19.5%), and SWS in 3 eyes (7.3%). Most patients were white Caucasian (13 patients, 61.9%) or black Afro-Caribbean (5 patients, 23.8%). Thirteen patients (61.9%) were male and the mean age was 41.3 ± 4.1 years at the last visit. In the PCG patients, the mean age at diagnosis was 1.1 ± 0.3 years.

**Table 1.** Patient demographics.

	Number (%)
*Primary diagnosis, eyes (n* *=* *41)*	
Primary congenital glaucoma	16 (39.0)
Aniridia	14 (34.2)
Anterior segment dysgenesis	8 (19.5)
Sturge-Weber syndrome	3 (7.3)
*Gender, patients (n* *=* *21)*	
Male	13 (61.9)
Female	8 (38.1)
*Ethnicity, patients (n* *=* *21)*	
White Caucasian	13 (61.9)
Black Afro-Caribbean	5 (23.8)
Asian	1 (4.8)
Mixed other	2 (9.5)
*Age (years), patients (n* *=* *21)*	
0–20	4 (19.0)
21–40	7 (33.3)
41–60	4 (19.0)
> 60	6 (28.6)

### Clinical features

At the last follow-up visit, 14 eyes (34.1%) had significant visual impairment with a best-corrected visual acuity of 6/36 or worse, and 13 eyes (31.7%) had a good best-corrected visual acuity of 6/9 or better (Table 2). Analysing the functional visual outcomes of individual patients, we found that 11 patients (52.4%) had vision of 6/12 or better in at least one eye, and 5 patients (23.8%) had vision of 6/60 or worse in both eyes. At the last visit, the mean IOP was 18.8 ± 1.4 mmHg and the mean cup-to-disc ratio was 0.6 ± 0.1. In terms of visual field assessment, the mean deviation was −6.8 ± 1.8 in eyes with good vision (*n* = 13 eyes).

**Table 2.** Clinical features (*n* = 41).

	Number of eyes (%)
*Best-corrected visual acuity*	
≤ 6/9	13 (31.7)
6/12–6/24	6 (14.6)
6/36–6/60	6 (14.6)
CF or HM	7 (17.1)
PL or NPL	1 (2.4)
Fixes and follows	2 (4.9)
Unable to fix and follow	2 (4.9)
Unable to assess	4 (9.8)
*Intraocular pressure (mmHg)*	
≤ 21	31 (75.6)
> 21	10 (24.4)
*Cup-to-disc ratio*	
≤ 0.6	12 (29.3)
> 0.6	17 (41.4)
No view	12 (29.3)
*Anti-glaucoma medications*	
Single agent	8 (19.5)
Two agents	5 (12.2)
More than two agents	10 (24.4)
No agent	18 (43.9)
*Types of medications*	
Beta-blocker eye drop	18 (78.3)
Prostaglandin analogue eye drop	14 (60.9)
Carbonic anhydrase inhibitor eye drop	13 (56.5)
Alpha agonist eye drop	6 (26.1)
Oral acetazolamide	2 (4.9)

**Figure 1.**
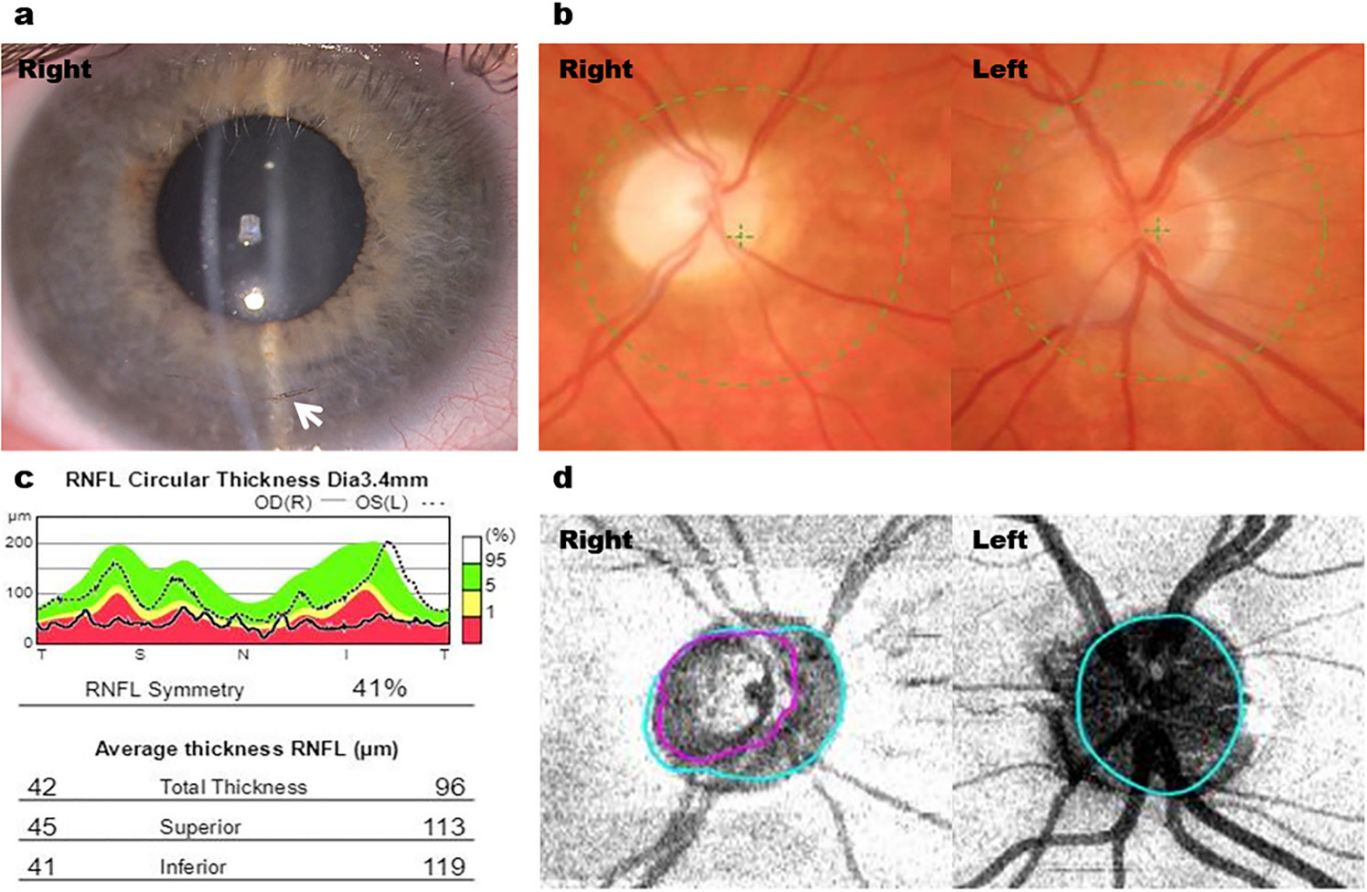
Right goniotomy in a patient with primary congenital glaucoma and advanced glaucoma. (a) Anterior segment photography of right buphthalmic eye with inferior Haab's striae (white arrow). (b) Fundus photography showing right pale cupped disc and left normal disc. (c) Retinal nerve fibre layer (RNFL) thickness showing right RNFL thinning (average thickness = 42 μm) compared to the left eye (average thickness = 96 μm). (d) OCT disc imaging showing marked cupping (purple line) of the right optic disc compared to the left normal optic disc.

**Figure 2.**
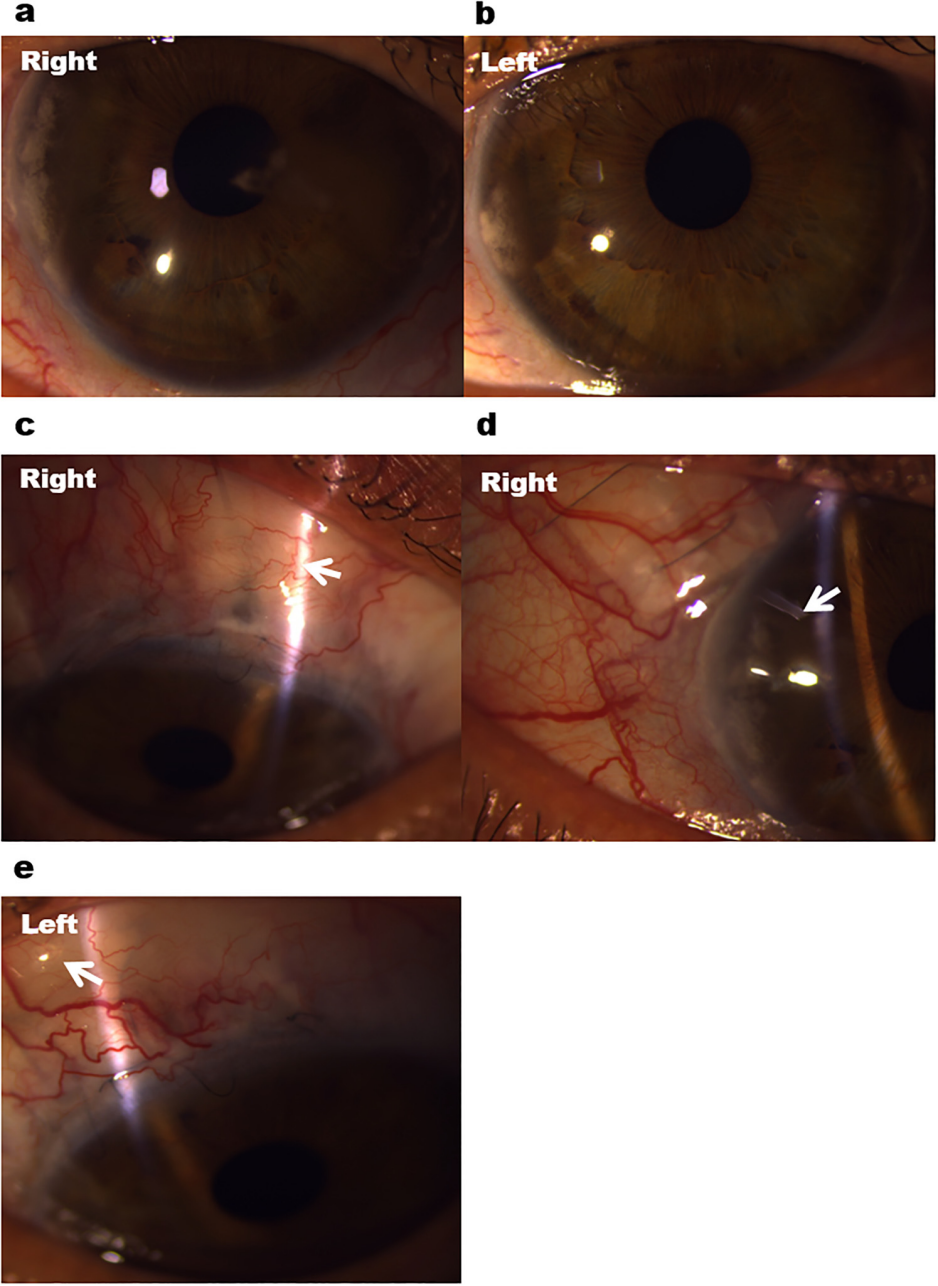
Multiple glaucoma surgeries in a patient with Axenfeld-Rieger anomaly and advanced glaucoma. (a, b) Anterior segment photography showing bilateral iris stromal hypoplasia and band keratopathy. (c, d) Failed scarred trabeculectomy bleb (white arrow) and subsequent Baerveldt tube surgery (white arrow) in the right eye. (e) Functioning trabeculectomy (white arrow) in the left eye.

**Figure 3.**
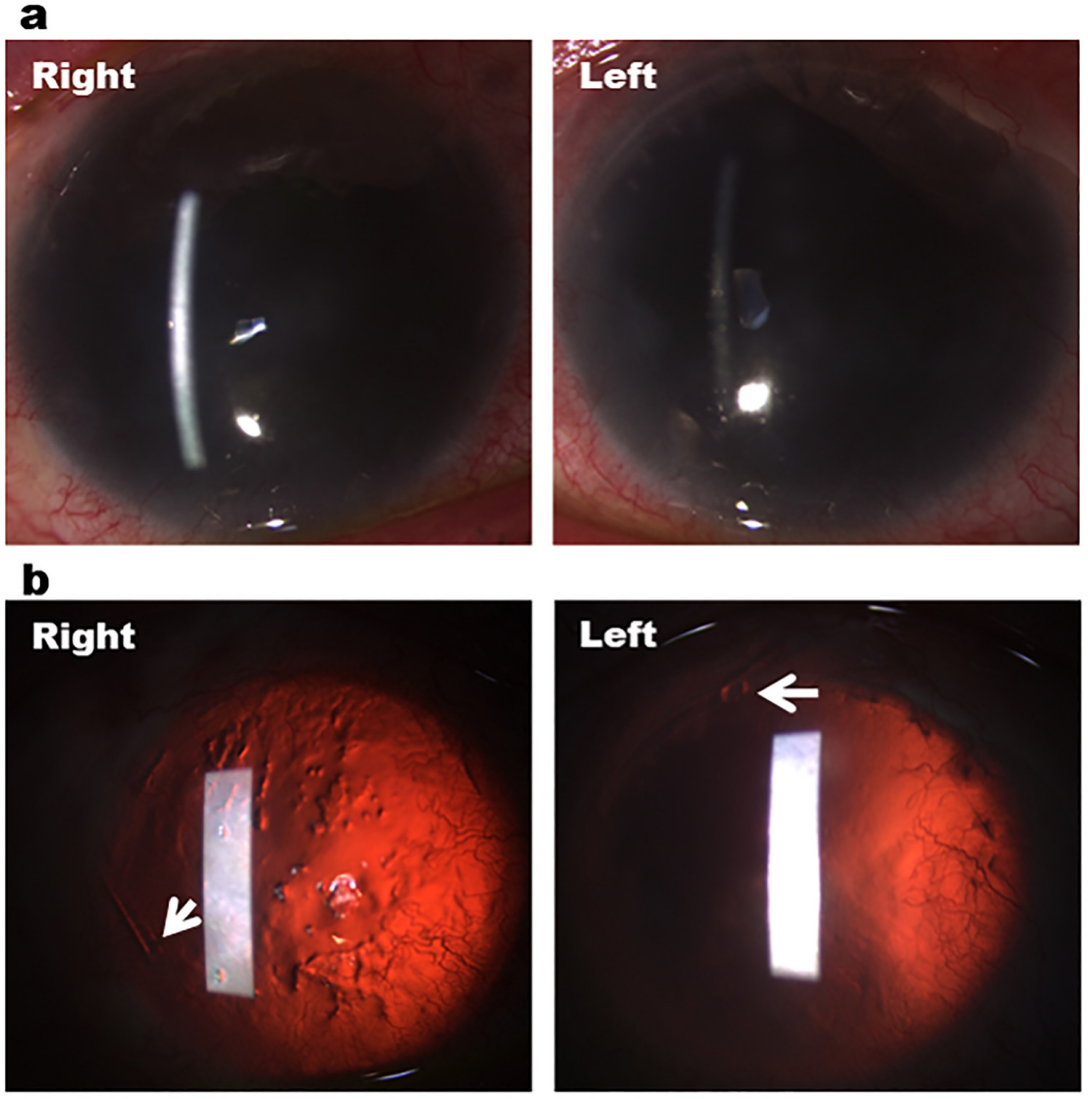
Bilateral baerveldt tube surgeries in a patient with aniridia and advanced glaucoma. (a) Anterior segment photography showing bilateral total aniridia and corneal neovascularisation. (b) Retroillumination showing total aniridia, 360 degrees corneal neovascularisation and right poor ocular surface. White arrows indicate the temporal tube position in the right eye and the superior tube position in the left eye.

With respect to clinical features, the patients with PCG displayed classical signs of buphthalmos, Haab's striae and disc cupping (Figure 1(a), 1(b)). The patients with Axenfeld-Rieger anomaly (AR) showed iris stromal hypoplasia, corectopia and band keratopathy (Figure 2(a), 2(b)), while those with aniridia had significant corneal vascularisation, nystagmus, subluxed lens and poor ocular surface (Figure 3(a), 3(b)).

### Medical management of glaucoma

Twenty-three eyes (56.1%) were on one or more anti-glaucoma medications to lower the IOP (Table 2). In terms of topical treatment, 18 eyes (78.3%) were treated with beta-blockers, 14 eyes (60.9%) with prostaglandin analogues, 13 eyes (56.5%) with carbonic anhydrase inhibitors, and 6 eyes (26.1%) with alpha agonists. One patient was also treated with oral acetazolamide.

### Surgical procedures

Sixteen eyes (39.0%) required one or more surgical or laser procedures for advanced glaucoma. Seven eyes (43.8%) had one surgery, 7 eyes (43.8%) underwent two surgeries, and 2 eyes (12.5%) needed more than two glaucoma surgeries. Goniotomy was performed in 9 eyes (56.3%) and trabeculotomy in 3 eyes (18.8%) of patients with PCG. Seven eyes (43.8%) also underwent trabeculectomy, namely 2 eyes with PCG, 2 eyes with aniridia, and 3 eyes with AR (Figure 2(c), 2(e)). Four eyes (25.0%) had insertion of Baerveldt tube, namely 1 eye with PCG, 1 eye with AR (Figure 2(d)), and 2 eyes with aniridia (Figure 3(b)). Cyclodiode laser was also performed in 2 eyes (13.0%) with PCG.

Of the 41 eyes included in our study, 19 eyes (46.3%) also had other ocular (non-glaucoma) surgeries, namely 13 eyes (68.4%) had cataract extraction with intraocular lens implant, 2 eyes (10.5%) had cataract extraction with no intraocular lens implant, 1 eye with aniridia (5.3%) had an artificial iris lens implant, and 3 eyes (15.8%) had vitreoretinal procedures such as vitrectomy and retinal laser.

### Surgical outcomes

**Figure 4.**
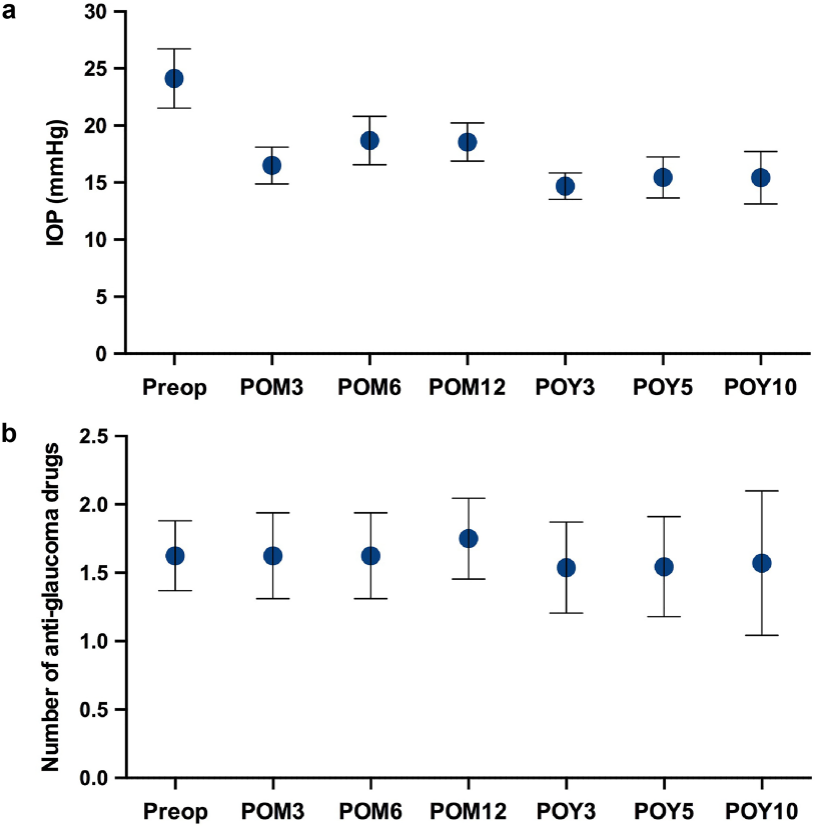
Long-term surgical outcomes in patients with advanced glaucoma. (a) Postoperative intraocular pressures (IOP) at 3 months (POM3), 6 months (POM6), 12 months (POM12), 3 years (POY3), 5 years (POY5) and 10 years (POY10) compared to preoperative IOP. (b) Number of postoperative anti-glaucoma medications compared to preoperative anti-glaucoma medications. Results represent mean ± SEM.

The long-term follow-up for the patients who underwent surgical or laser treatment for advanced glaucoma was 12.8 ± 3.6 years. There was a significant decrease in postoperative IOP (mmHg) at 3 months (16.5 ± 1.6; *p* = 0.0067), 6 months (18.7 ± 2.1; *p* = 0.0386), 12 months (18.6 ± 1.7; *p* = 0.0229), 3 years (14.7 ± 1.2; *p* = 0.0126), 5 years (15.5 ± 1.8; *p* = 0.0330) and 10 years (15.4 ± 2.3; *p* = 0.7780), compared to preoperatively (24.1 ± 2.6) (Figure 4(a)). We found that the non-PCG group had a larger decrease in mean IOP preoperatively to postoperatively, 25.0 to 13.0 mmHg, compared to 29.5 to 20.5 mmHg in the PCG group. The PCG group also had a longer follow-up period of 16.9 ± 5.6 years compared to 7.2 ± 2.8 years in the non-PCG group.

**Figure 5.**
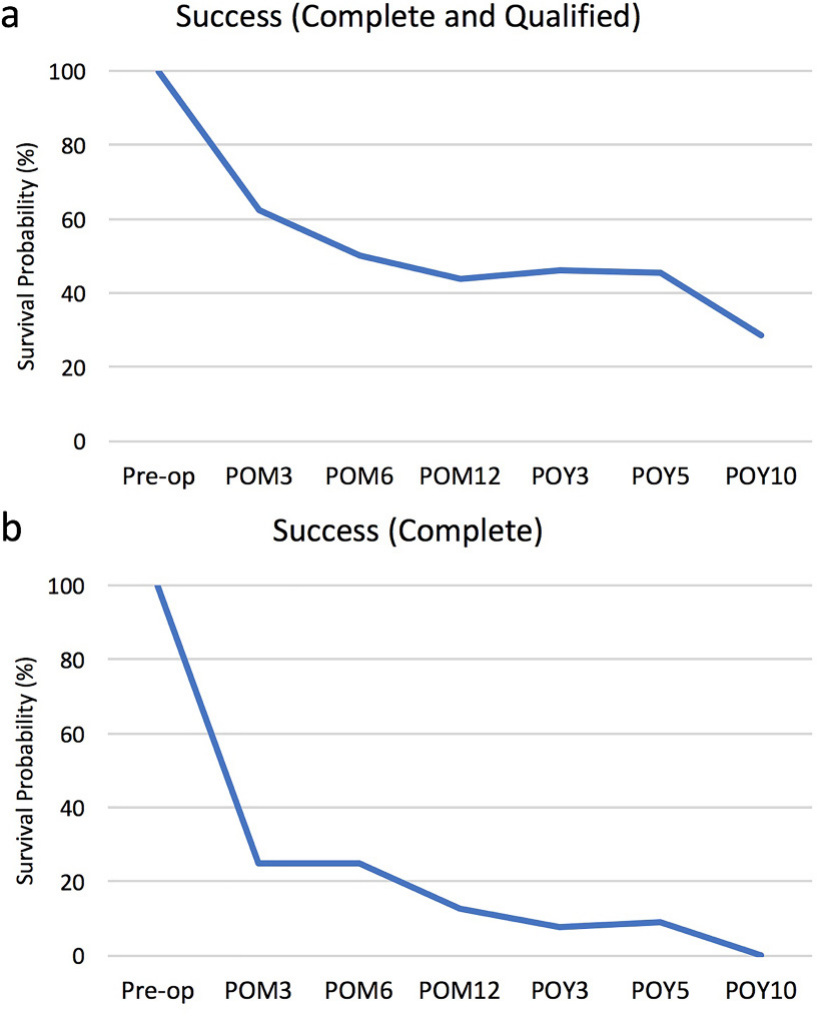
Kaplan Meier survival probability graphs for (a) combined complete and qualified success and (b) complete success only at 3 months (POM3), 6 months (POM6), 12 months (POM12), 3 years (POY3), 5 years (POY5) and 10 years (POY10) postoperatively.

There was no significant change in the number of anti-glaucoma medications postoperatively at 3 months (1.63 ± 0.31; *p* = 0.9999), 6 months (1.63 ± 0.31; *p* = 0.9999), 12 months (1.75 ± 0.30; *p* = 0.6524), 3 years (1.54 ± 0.33; *p* = 0.9999), 5 years (1.55 ± 0.37; *p* = 0.7787) and 10 years (1.57 ± 0.53; *p* = 0.3559), compared to preoperatively (1.63 ± 0.26) (Figure 4(b)). In terms of Kaplan Meier graphs, survival probability for combined complete and qualified success was 62.5%, 50.0%, 43.8%, 46.2%, 45.5% and 28.6% at 3 months, 6 months, 12 months, 3 years, 5 years and 10 years, respectively (Figure 5(a)). Survival probability for complete success only was 25.0%, 25.0%, 12.5%, 7.7%, 9.1% and 0.0% at 3 months, 6 months, 12 months, 3 years, 5 years and 10 years, respectively (Figure 5(b)).

### Surgical complications

Of the 16 eyes who underwent one or more surgical or laser procedures, one eye (6.3%) lost > 2 Snellen lines at 3 months follow-up and three eyes (18.8%) lost > 2 Snellen lines at 6 months follow-up. There were no reported intraoperative complications in these complex surgery eyes. Four eyes (25.0%) had postoperative complications. There was one case of hyphaema after trabeculotomy in a patient with PCG, which reabsorbed after a few days. Three eyes (2 eyes with aniridia after Baerveldt tube surgery and 1 eye with AR after trabeculectomy) also had persistent hypotony (IOP ≤ 5) but deep anterior chamber and no choroidal detachment or hypotonous maculopathy, and were managed conservatively with no complications.

## Discussion

This study describes the long-term surgical outcomes of a cohort of complex glaucoma patients with PCG, aniridia, ASD and SWS in two UK tertiary referral centres. The most common condition in the cohort was PCG (39.0%). PCG has a predominantly autosomal recessive inheritance pattern with a polygenic aetiology, and *CYP1B1, LTBP2, MYOC, FOXC1* and *TEK* genes have been involved in the pathogenesis of this condition.^10^ This condition develops from isolated trabeculodysgenesis, commonly presents with epiphora, photophobia and blepharospasm, and can affect one or both eyes.^11^ The patients with PCG in our cohort presented with classical signs of buphthalmos, Haab's striae, raised IOP and optic disc cupping.

The BIG Eye Study studied 99 patients with newly diagnosed childhood glaucoma in the UK, with 47 patients having PCG and 52 patients having secondary glaucoma.^2^ The BIG Eye Study found the highest incidence of PCG amongst children of Pakistani origin, which was nine times that of white Caucasians, followed by Bangladeshis and Indians. Interestingly, in our cohort of patients with PCG in South London, 37.5% were black Afro-Caribbean, 37.5% were white Caucasians, 12.5% were Asians, and 12.5% were mixed. These differences could be explained by regional differences in ethnicities as about 20% of the population in London are of black Afro-Caribbean origin. In terms of gender distribution, 61.9% of patients in our study were male and 38.1% were female, which was similar to the 1.5:1 male to female distribution of secondary glaucoma described in the BIG Eye study.^2^

Aniridia was the second most common condition, accounting for 34.1% in the cohort. Aniridia is a rare condition which has a propensity to induce secondary glaucoma, with a reported incidence ranging from 1: 64,000 to 1: 96,000.^12^ It is characterised by bilateral absence of the iris but there is significant variation amongst those affected. Aniridia also affects other structures, such as the optic nerve, retina, lens and cornea. Systemically, it is associated with Wilms’ tumour with a reported incidence of 1%,^13^ genitourinary abnormalities and mental retardation.^14^ Aniridia has been linked to a mutation in the *PAX6* gene on chromosome 11p13.^15^ Generally, inheritance is in an autosomal dominant pattern and homozygous expression is lethal in utero.^16^ Clinically, aniridia presents with reduced vision due to foveal maldevelopment. Patients also suffer progressive sight loss from glaucoma, corneal opacification and cataracts.^12^ The patients with aniridia in our cohort had significant visual impairment with corneal vascularisation and opacification, advanced glaucoma, marked nystagmus and cataracts.

ASD describes a group of conditions that impact ocular development. AR is a type of ASD affecting the development of the iris, cornea and lens. Within our patient cohort, 19.5% were affected by ASD. ASD is a rare autosomal dominant condition with an estimated prevalence between 1: 50,000 to 1: 100,000.^17^ Frequently, affected individuals present with elevated IOPs owing to abnormalities in the iridocorneal angle, which can affect aqueous drainage. These patients are thus at higher risk of developing glaucoma with as many as 50% of patients being affected.^17^ The anterior segment is derived from surface ectoderm, neural ectoderm and periocular mesenchyme. Disruption or imbalance of developmental genes and signalling pathways results in this miscoordination of embryonic development. Defective migration or differentiation of mesenchymal cells has been highlighted as a cause of ASD.^18^

The smallest group of affected eyes (7.3%) in this study were those patients affected by SWS. It is a rare condition affecting the brain, skin and eyes with a reported incidence of 1: 50,000 infants.^19^ SWS is characterised by a triad of facial port wine stain, leptomeningeal capillary-venous malformation and ocular defects.^20^ It is associated with a somatic mutation in the *GNAQ* gene, resulting in the development of hamartomas.^21^ The most frequent ocular comorbidity is glaucoma with a prevalence of 30–70%. The age at which glaucoma presents has a bimodal distribution, affecting 60% of patients congenitally and 40% later in their childhood or adolescence.^20^ There are multiple proposed mechanisms for the development of glaucoma in SWS, one of which is an increase in episcleral venous pressure, leading to the obstruction of aqueous outflow. In our cohort, one patient also had a choroidal haemangioma which is often a component of SWS.^22^ Many patients with SWS also experience cognitive problems and learning impairment, and one patient in our study had marked learning difficulties.

In childhood glaucoma, surgery is the mainstay of treatment and angle surgery is the most frequently performed procedure.^23^ In the Big Eye Study, 94% of eyes with PCG and 64% of eyes with secondary glaucoma underwent surgery to control the IOP.^2^ In our cohort, a lower percentage of eyes (39.0%) underwent glaucoma surgery, with goniotomies constituting 56.3% of the procedures, trabeculotomies 18.8%, trabeculectomies 43.8%, and Baerveldt tubes 25.0%. However, out of the eyes that were operated on, 56.3% needed multiple glaucoma surgeries to control the IOP. The lower number of surgically managed cases in our study could be explained by our cohort consisting of a high number of eyes (*n* = 25, 61.0%) with a primary diagnosis of aniridia, ASD and SWS with adult onset glaucoma, that were hence managed medically. Childhood glaucoma is difficult to treat and patients often require multiple procedures during their lifetime. Moreover, the hospitals involved in this study are tertiary referral centres and the patients referred often have complex pathologies. Overall, the patients in the cohort had good surgical outcomes with a significant decrease in postoperative IOP by 36.1% compared to preoperative IOP, and no significant change in the number of anti-glaucoma medications after long-term follow-up.

This study contributes to the long-term surgical outcomes of a complex group of sight-threatening childhood glaucoma conditions worldwide. The limitations are that it is a retrospective study and cases were collected from two tertiary centres in the UK. Owing to the rarity, there are only a small number of published cohorts and possible comparisons between studies are therefore limited. Surgical management of these complex eyes with advanced glaucoma is often challenging, and requires an experienced surgical and multidisciplinary team to maximise the chance of surgical success and good long-term visual outcome.
